# Modern Alterations in the Matron's Position

**Published:** 1907-04-13

**Authors:** 


### MODERN ALTERATIONS IN THE MATRON'S POSITION.

With the growth of the modern hospital the position of the superintendent of nurses is becoming modified by successive small changes until she finds herself confronted with duties which tax all the powers of an exceptionally able woman. That these posts carry a salary out of all proportion to the weighty responsibilities with which they are charged is no bar to their being earnestly desired by those best qualified to fill them. And if, as Aristotle taught, happiness is only to be attained in this world "through the exercise of our highest faculties," it is not, perhaps, surprising that women are found gladly to devote the best years of their lives to labours which give scope for the exercise of moral and intellectual qualities of no common kind.

In old days the matron took an intimate and practical share in the actual nursing. She would often devote herself to a critical case, and no operation took place without her attendance. The modern matron refrains with religious exactitude from performing nursing duties under any circumstances, much as she may hunger to be using her skill; and should she attempt to be present in each operating-room whenever it is used, a hundred other duties would perforce be neglected. She is charged with the responsibility of bringing the nursing to the highest pitch of excellence, and what is even more difficult, of maintaining it there through successive years; her observation of all that passes in the wards and in the theatre is unceasing, and her influence all pervading; but she works through others, and to the patients is little more than a kindly presence with a wonderful memory for their symptoms, perhaps, but no mantle of conscious authority such as their sister wears becomingly. It is not for them to know that the reason why the junior probationer's eyes are so red is because matron said a word to sister on her way out of the ward after one of those brief visits which leave the impression that she is merely on her way somewhere else.

For the times have changed as regards the matron's relations to her staff. The tender and intimate association of the old matron with the nurses who were trained under her wing can hardly be maintained with the big development of the training school. The probationer must look to her ward sister for rebuke and encouragement alike. She must depend on the home sister for advice in her affairs and for the friendly control which to girls fresh from a quiet home life is so often valuable. The matron knows every step of their career through the hospital, and can measure their worth to an ounce; but they do not know, and never will know unless there is the gravest reason for bringing it under their notice the extent to which she is learning to gauge their character and powers. A great reserve force in the background—that is the position of the matron of to-day with regard to her probationers.

Still more has her position changed with regard to her sisters. How the old sisters stayed on and on. Doctors came and went, and matrons had their day; and yet the veteran sisters, who in the eyes of the students stood almost for symbols of the mother hospital herself, were found year by year unchanged and defying time. Everyone regrets the old sisters, and where they still linger the heart goes out to them as to the very best product of a passing system. But passing they are. In the present day the sister is learning experience. She may stay three years, she may even stay ten, but she is a bird of passage, and sooner or later she gets promotion or starts private nursing, and her place in hospital knows her no more. In consequence, the modern matron is for ever in process of training her sisters. There are no longer stereotyped ways in particular wards. The fresh air of recent theories is blowing in and out everywhere, and blows nowhere so hard as among the modern sister's duties, inspired by the modern matron. It gives a great deal more trouble, but the modern matron likes it. If her heart fails her at the resignation of one on whom she has learned to depend, the next minute, be sure, she is planning some alteration which she could only carry out with an entirely fresh hand. There are always compensations in hospital life.

What shall be said of the matron's part in that complicated machinery outside the wards and the training school which runs the hospital of modern times? Her staff of servants, continually changing in accordance with the sense of fluctuation in the institution, is in itself a serious charge. Constantly engaging, constantly bringing into shape, and when they leave to "better themselves," constantly giving characters, the matron can never sit down as her predecessor used to do in the comfortable assurance that only old retainers or their wellrecommended daughters made up her domestic staff. There are days off and nights out, and time-tables to be modified in accordance with the domestic catastrophes which are the rule rather than the exception in hospital life, and all enormously complicated by modern and wiser notions of the amount of recreation due to young servants. It is the matron who keeps these strands in her hands, even when she has the aid of a housekeeper, for it is on the matron's knowledge of character and on her judgment which the housekeeper relies in the superintendence of the big domestic staff.

Many of the duties which in old time fell to the matron have now been shifted from her overburdened shoulders. The accounts are rarely assigned to her, though this used to be a very common practice, and has only recently, in fact, been abandoned by King's College Hospital. Her only share in bookkeeping is in regard to her petty cash, and this has been brought down to such small dimensions in some institutions that the matron's only use for money is to buy her weekly store of stamps. All this is clear gain, and there was need for relief, since manifold departments, laundry, linen-room, stores, dressings, have come under her sway, in all of which initiation has taken the place of routine.

There is one word which sums up the modern matron's duties, and that is "supervision." In that is included all her talents; it is the secret of all her success. She dare not settle down to spend long hours in her office, no matter what important duties she may be performing there. She is bound to devote her time to incessant and vigilant observation of every department under her care; she is always active, never hurried. Her brain is incessantly recording minute facts, and each fact calls for some action on her part or that of another. She has an admirable system for the distribution of her time, yet every moment of her day is at the mercy of events outside her reckoning. Amidst this unceasing strain on brain and temper one thing tends to keep her equable, and is, in fact, responsible for the excellent health commonly enjoyed by the modern matron. She walks on a moderate computation at least ten miles a day, and whatever else she may be deprived of in the busy life she has chosen, it is certainly neither air nor exercise.

